# Phase-Amplitude Coupling Localizes Pathologic Brain with Aid of Behavioral Staging in Sleep

**DOI:** 10.3390/life13051186

**Published:** 2023-05-15

**Authors:** Brent Berry, Yogatheesan Varatharajah, Vaclav Kremen, Michal Kucewicz, Hari Guragain, Benjamin Brinkmann, Juliano Duque, Diego Z. Carvalho, Matt Stead, Gary Sieck, Gregory Worrell

**Affiliations:** 1 Department of Neurology, Mayo Clinic, Rochester, MN 55905, USA; 2 Department of Physiology & Biomedical Engineering, Mayo Clinic, Rochester, MN 55905, USA; 3 Biomedical and Electrical/Computer Engineering, University of Illinois at Urbana-Champaign, Urbana, IL 61801, USA; 4 Czech Institute of Informatics, Robotics and Cybernetics, Czech Technical University, 160 00 Prague, Czech Republic; 5 Department of Computing and Mathematics, FFCLRP, University of São Paulo, Ribeirão Preto 14040-901, SP, Brazil

**Keywords:** behavioral staging, phase-amplitude coupling (PAC), epilepsy

## Abstract

Low frequency brain rhythms facilitate communication across large spatial regions in the brain and high frequency rhythms are thought to signify local processing among nearby assemblies. A heavily investigated mode by which these low frequency and high frequency phenomenon interact is phase-amplitude coupling (PAC). This phenomenon has recently shown promise as a novel electrophysiologic biomarker, in a number of neurologic diseases including human epilepsy. In 17 medically refractory epilepsy patients undergoing phase-2 monitoring for the evaluation of surgical resection and in whom temporal depth electrodes were implanted, we investigated the electrophysiologic relationships of PAC in epileptogenic (seizure onset zone or SOZ) and non-epileptogenic tissue (non-SOZ). That this biomarker can differentiate seizure onset zone from non-seizure onset zone has been established with ictal and pre-ictal data, but less so with interictal data. Here we show that this biomarker can differentiate SOZ from non-SOZ interictally and is also a function of interictal epileptiform discharges. We also show a differential level of PAC in slow-wave-sleep relative to NREM1-2 and awake states. Lastly, we show AUROC evaluation of the localization of SOZ is optimal when utilizing beta or alpha phase onto high-gamma or ripple band. The results suggest an elevated PAC may reflect an electrophysiology-based biomarker for abnormal/epileptogenic brain regions.

## 1. Introduction

Epilepsy is a chronic disease which is defined by recurrent seizures [1]. Approximately 1/3 of patients are not amenable to conservative treatment but can be evaluated for surgi-cal resection of the pathologic brain tissue once failing enough anti-seizure medications (ASMs). The purpose of this surgery is to remove the seizure onset zone (SOZ) which is defined as the area of cortex from which the seizures originate [2,3]. The gold standard method for the evaluation of SOZ is ictal recording from intracranial electroencephalo-gram (iEEG) and synchronized video [3,4,5]. Patients are implanted with multiple electrode arrays in this process and monitored in the ICU as seizures can take days or weeks to oc-cur. During this time, a great deal of interictal data are also recorded but are of less use to the clinical teams. However, much work has been done on this interictal data in both the human cognitive domains and epilepsy domains, looking for information on normal and abnormal human brain physiology [6,7,8,9]. Among the many electrophysiologic biomarkers being investigated [10,11], phase-amplitude coupling is one which has emerged as a reasonable promising marker to be used in seizure prediction [12] and detection [13] as it may reflect a perturbation of underlying normal physiology. Interictal evaluations have involved largely cognitive or behavioral testing [14,15,16] and have made assumptions that any electrodes in the seizure onset zone are of little consequence for analysis. The aim of this report is to investigate the role of seizure onset zone electrodes as it relates to phase-amplitude coupling (PAC).

Greater insights into mechanisms of large neuron population behavior and the brain oscillations emanating therein may not only improve seizure onset localization and sei-zure prediction, but also help improve information surrounding the pathophysiology of seizure initiation in the brain [12,17,18]. Local field potentials made with iEEG relating to seizure activity are shown to be confined to millimeter scales on the brain [12]. High-frequency activity (HFA) which includes gamma oscillations and ripples (up to 500 Hz) have been associated with epileptiform activity in human epilepsies [18,19,20].

The cortex has natural brain rhythms ranging over five scales of magnitude [21]. There is a logarithmic inverse relationship between power and frequency, known as power-law, where power drops off from low frequencies to high resulting in an approximate 1/f rela-tionship rather in lower frequency range 0–40 Hz [5,22]. This is a signature of scale-free sys-tems [22] where higher frequencies are modulated by lower frequencies (in phase-phase or phase-amplitude coupled scenarios); larger networks are recruited by lower frequencies [21]. There is evidence for these dynamics both physiologically and pathologically [23,24]. These processes have been investigated in terms of infraslow relationship (<0.1 Hz) to interictal epileptiform spike trains, but not within standard Berger Band ranges as it relates to HFA.

In the temporal lobe, PAC between theta phase and high gamma amplitude is ob-served largely [19,25]. In different anatomical areas, it would not be expected that theta-gamma is the relevant cross frequency coupling of oscillations. Beta-gamma between thalamus and motor cortex or alpha-gamma [26] in the occipital lobe [14] has been shown. The relevant cross coupling has also been shown to be behavioral state dependent. Slow wave sleep particularly has been shown as relevant for cross frequency coupling [27]. Edakawa showed that seizures can be detected using PAC when utilizing beta and high gamma as an ad-junct to ictal biomarkers [13]. Their work alluded to the possibility of interictal periods where synchronization index (a specific kind of PAC described in Osipova) values increase and may generate false-positives in differentiating ictal from interictal state, but whether long periods of interictal time could be used to differentiate SOZ from non-SOZ was not a ques-tion explored. Little work has been done on determining the best frequency range and best behavioral state in interictal time to determine the seizure onset zone or whether even seizure onset zone can be determined using PAC as an interictal biomarker.

Coupling between slow potentials and high frequency activity as a phenomenon is being actively studied in many diseases and in normal physiologic states [28,29,30]. How this phenomenon relates to epilepsy is very much an open question—insights can be gained into cortical network excitability [12]. It has been shown that slow oscillations are able to trigger and group HFA. The coupling between Berger bands and HFA has been termed nested oscillations and may be significant signature of cortical activation and perhaps a novel biomarker in epilepsy [31,32,33]. Previous studies in iEEG have identified PAC of HFA (40–180 Hz) modulated by theta or delta (0.5–9 Hz) [12]. These observations helped develop a notion to study in detail the physiology of PAC in patients with medically refractory focal epilepsy.

Phase-amplitude coupling’s physiologic role as it relates to information transfer re-quires a precise temporal structure. This structure, governed by natural behavioral pat-terns such as sleep, has to be provided by the brain activity itself. Non-rapid eye move-ment sleep (NREM) is characterized by three neuronal oscillations that provide a scaffold for information transfer. Beyond spindles or k complexes (features of N2) are slow oscilla-tions around 0.5–1.0 Hz, a feature of N3 [34]. These reflect widespread variations in cellular excitability resulting from interchanging phases of hyperpolarization and depolarization of large neuronal pools [35,36]. Slower oscillations traverse most of the neocortex, hippocampus, and thalamus, from which sleep spindles arise [37,38]. Normal physiology of information transfer supported by PAC involves slow oscillations coupled with sleep spindles, which are in turn supported by coupling to high gamma or ripple bursts. In human epilepsy however, it has been postulated that this normal physiology is disrupted in seizure onset zones [28,39,40]. To date, little work has been carried out exploring PAC in the context of sleep and epilepsy. Here we investigated how different sleep states change the characteristics of PAC in seizure onset zone and non-seizure onset zone and how we can use this information to automatically find SOZs within interictal periods during sleep.

The “active consolidation” theory details information transfer in the brain being supported by these physiologic phenomena [41]. The depolarizing state enables sleep spin-dles which themselves enable ripple at least in limbic circuitry. This is thought to function in the neocortical-hippocampal LTP problem. The “active consolidation” basis of sleep details that there is a transfer or reinforcement of percepts which relies on the interplay of slow oscillations, specifically the up-state at which time spindles emerge and then ripples within those spindles represent discrete units of information. All of this occurs in the temporal lobe and information is theorized to be disseminated or linked to the neocortex for permanent or near-permanent storage. This theory implies that ripples and spindles are linked at the hippocampus between thalamus and neocortex. With iEEG, it is possible to measure these phenomena [37,38] and it is plausible that this particular physiologic func-tion is perturbed in epilepsy, particularly temporal lobe epilepsy [42].

Timing precision is critical for information transfer in the brain [21] and if such transfer occurs during sleep then the coupling of waves with different wavelengths can be ex-plored in support of such a theory. Slow oscillations (sub-delta) are believed to reflect global fluctuations in cellular excitability where each up-state is hyperexcited relative to the down-state [27]. These slow oscillations emerge spontaneously in frontal lobe. These waves are believed to be involved in spatially distributed neocortical and hippocampal and thalamic circuitry and are believed to be causal with spindles. Spindles themselves at ~15 Hz originate at thalamic and reticular activating system generators. There are projec-tions to neocortex and hippocampus. Ripples are high frequency oscillations which have been explored in humans at around 100 Hz [10].

There has been little investigation into the relationship between interictal epilepti-form discharges or spikes and phase-amplitude coupling. Burgess describes interictal in-fraslow waves with “nested oscillations” or “spike-crested oscillations” which may rep-resent a phase-coupled relationship; these efforts have largely been qualitative to date [43,44]. Weiss et al. describe ripples on spikes as PAC in SOZ of mesial temporal lobe patients but did not explore the sensitivity and specificity of such measures in determining SOZ in a large cohort or explore other frequency–phase relationship sensitivity/specificity metrics or behavioral state dependence [18].

Previously interictal epileptiform discharges (IED) and high frequency oscillations (HFO) have been explored as interictal electrophysiologic biomarkers [16,45], but more recent-ly phase-amplitude coupling (PAC) has been investigated and shown preictally to localize epileptogenic tissue and also to detect seizures [12,13,17]. Whether PAC can be used interictally is unclear and under what parameters and behavioral states investigation should proceed is an open question [28,29] but has been attempted to be addressed by other studies which have overlapped with this study [27]. Here we use a modified detection method with improved sensitivity to analyze PAC on ten medically resistant epilepsy patients whose 2 h night segments of iEEG data were analyzed. These patients also had simultaneous scalp EEG recording that was used to score behavioral stages according to AASM 2012 standards. The following questions are addressed here.

The phase of what frequencies are modulating the amplitude of which bands and are relevant in discriminating SOZ from non-SOZ is an open question. During which behavioral (sleep) states is this PAC observed and relevant for epileptogenic (SOZ) brain is another question. To what extent is PAC a reflection of other biomarkers (HFO, IED) is finally another question which is being investigated herein.

## 2. Materials and Methods

### 2.1. Subjects

Per Institutional Review Board protocol, 17 patients who were under evaluation for resective surgery for medical refractory epilepsy (MRE) at Mayo Clinic in Rochester MN were included in this study. Institutional Review Board (IRB) approved the study and necessary consenting procedures were followed prior to any data acquisition. All subjects had bilaterally placed intracranial depth electrodes with usually eight contacts to record intracranial electroencephalography data (iEEG). In some cases, not all contacts could be used for data acquisition (hardware or recording problems). Six subjects of this cohort had simultaneous scalp EEG, EOG, and EMG recordings placed for the purpose of gold standard sleep scoring. Subjects 1–10 were the subjects of detailed analysis on spike count, HFO, PAC as in Figure 5, but for subjects 11–17, sEEG patient was analyzed but not included in the final AUROC analysis of behavioral states in Figure 6. Subject recordings were ignored for post operative day (POD-1) as anesthetics were dissipating. Subjects then stayed in the ICU ranging from 3–12 days before explanation.

### 2.2. Data Acquisition

Data were acquired using a reference placed at the vertex of the scalp between Cz and Fz. This acted to electrically isolate the recording electrodes which had several centimeters of muscle and bone between reference and recording. This distance requires a large ~3 × 3 cm cortical source to generate a potential change on the reference electrode. Practically speaking, however, a scalp reference is diminutive relative to a subdural or depth intracranial electrode. The iEEG and scalp EEG, EOG, and EMG data were acquired with a DC-capable Neuralynx electrophysiology system. iEEG data were digitized at 32 kHz (EEG, EOG, and EMG at 1 kHz). For analysis in this paper, bipolar iEEG was analyzed, the iEEG data were offline filtered by 1 kHz low-pass filter (Butterworth 3rd order) and decimated down to a frequency of 5 kHz. A notch filter for line noise was not applied. Scalp EEG, EOG, and EMG data were filtered by 250 Hz low-pass filter (Butterworth 3rd order) and downsampled to 500 Hz.

### 2.3. Contact Placement and Co-Registered Localization

**Figure 1:**
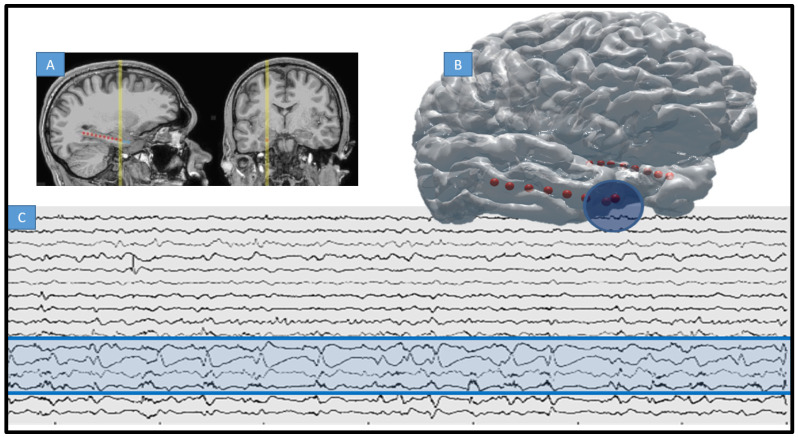
Intracranial EEG and MRI-CT co-registered electrodes. (**A**) T1 showing location of 4 macroelectrode contacts implanted along the hippocampus of a patient with MTS and MRE of temporal lobe (arrow points to electrode trajectory indicating occipital approach). (**B**) 3D rendering of typical electrode placement within same patient, red indicating macro contact of depth and blue the particular SOZ for this patient (first four electrodes of right depth, or anterior temporal pole). SOZ = seizure onset zone. (**C**) Raw intracranial EEG trace for same patient are recorded on 8 depth electrodes as in (**A**) (SOZ marked in blue circle and blue shading on raw EEG).

Depth electrodes (AD-Tech Medical Inc. Oak Creek WI) consisted of a 1 mm diameter polyurethane shaft with platinum/iridium (Pt/Ir) clinical macroelectrode contacts spaced ~5 mm from each other. Impedances were measured from 50–150 kΩ. Identification of implanted contacts was carried out post-implantation using co-registered CT/T1 brain images (CT post-op and MRI pre-operatively acquired). Co-registered images were modeled onto a MNI brain space and resulting coordinates were mapped for each patient. This was carried out using the 3-D BRAIN ANALYZE software (Analyze Direct, Inc. Minnetonka MN), and a model of electrodes was reconstructed within the MRIcroGL brain imaging software (McCausland Center for Brain Imaging) using transparent brain template ch256. Anatomical localization of electrodes was achieved by co-registering post-implant CT data and co-registered to the patient’s high resolution MRI using a normalized mutual information algorithm (SPM8, Wellcome Trust Centre for Neuroimaging); mutual information modalities have been used with success previously in the co-registration problem. Normalization to MNI was done using an affine transformation followed by a smoothing basis function and then manual identification of electrodes relative to specific anatomies was performed (see Figure 1). Patients in this study have a similar electrode layout given a similar neurosurgical approach and thus anatomies can be considered a constant [46]. This entire process is not automated but semi-automated; Dr. Brinkmann manually identified the outputs from the above-described processes as part of the clinical workflow.

#### 2.3.1. Artifact Identification and Night Segment Data Choice

Scalp and iEEG recordings from each subject were reviewed using a MATLAB viewer created by Mayo System Electrophysiology Lab Version 1.0 [47]. Seizures, artifacts, and discontinuities were identified and only 2-h night segments (either 12 a.m. to 2 a.m. or 1 a.m. to 3 a.m.) that were free of such signal debris were utilized in this study. Another analysis with IEEE examined varying night epoch segment length. Furthermore, to detect multiple sleep stages would require more than 30 min data segment analysis. Moreover, no chosen data segment was within 3 h of a seizure. Interictal spikes were not considered artifacts and were, in fact, catalogued for analysis.

#### 2.3.2. Interictal Epileptiform Discharge and High Frequency Oscillation, and Phase-Amplitude Coupling Identification

**Figure 2:**
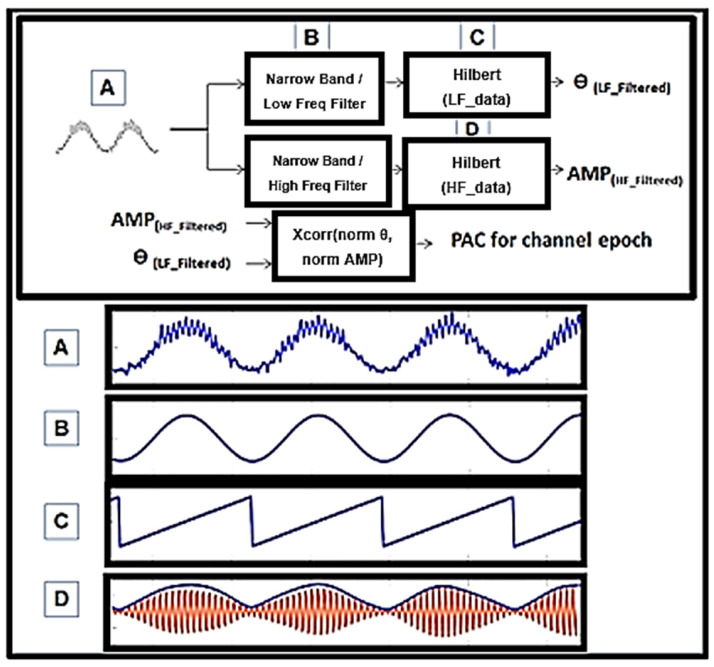
Phase-amplitude coupling on example 3 s synthetic epoch. (**A**) A signal was synthesized and combined between two differing amplitude and frequency sine waves. (**B**) Input from (**A**) in this detector is filtered using wavelets width = 9. This is not the only way to perform this calculation but it is the one utilized here. Center frequency was measured at 2 Hz spectral bins and midpoint f. (**C**) Angle (LF_filtered) was utilized where instantaneous phase, ϴ, of (**B**) could not be compared to (**D**). (**D**) The envelope of the instantaneous power of HF_filtered, AMP, was extracted and correlated with C. Xcorr, coherence, and simple Pearson’s correlation were utilized.

Using a previously validated interictal epileptiform discharge (IED) detection algorithm which was developed in Matlab [48] and can be used on .mef compression format, spike detection was performed on all data segments, and timestamps of detected events can be stored to MySQL (see Supplement File S1). Initial development of this algorithm was carried out on training patients and was independent of testing on which the statistical analysis was performed with final input from an epileptologist not involved in the marking of testing data [48]. High frequency oscillation detection was undertaken also using previously validated methods [16,49]. HFOs were detected using a Hilbert transform-based method, as previously reported. Here, the discrete time series is transformed into an analytic signal, where the real part is the original signal, and the imaginary part is the Hilbert transform of the original, x(t). See Appendix A for more information on PAC calculations; the basic framework is in Figure 2.

The data segments were bandpass filtered (Butterworth, third order) for every 1-Hz band step from 50 to 500 Hz. Then, the filtered-data frequency bands were independently z-scored to obtain a power-change estimate and plotted together on a time-frequency spectrogram [15]. Incidences of significantly increased power on the resultant spectrograms were determined by transforming the time-frequency power changes into a series of binary values, where “1” signified a threshold of greater than 3.0 z-score. Only detections with a minimum of one complete cycle above the threshold were included in the analysis. The threshold was set at the same value as in the previous studies [10].

**Figure 3:**
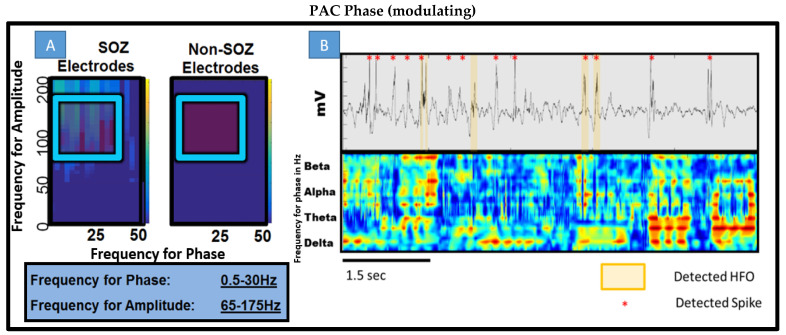
Phase-amplitude coupling gram (PACgram) and overlay of PAC vs. time plot with raw EEG and spike detection. (**A**) Utilizing PAC detection, 3 s epochs, non-overlapping windows. Collection of all SOZ electrodes in patient 7 and non SOZ electrodes for a 1 min epoch. According to the previous literature, 0.5–30 Hz for phase with gamma and ripple frequency for amplitude noted in blue box was the starting point for all further experiments. (**B**) *Top* Raw EEG tracing across ~6 s with both HFO displayed and detected spikes during a SWS epoch. *Bottom* Moving window 50 ms overlapping calculations of PAC for different bands for phase on constant-held amplitude of 65–175 Hz. This represents the design of the experiment elucidating which bands are contributing to composite PAC values. The data are taken during a SWS segment from patient 8. Note the strength of delta on gamma/ripple and beta prevalence.

**Figure 4:**
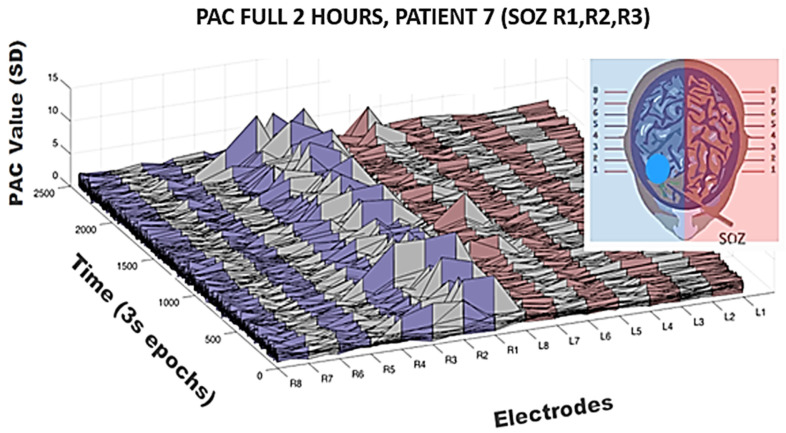
Temporal variation of the feature PAC on all the channels in seventh patient. Log-normalization performed on composite PAC values between a wide low frequency modulating band (0.5–30 Hz) onto a wide high frequency band (65–175 Hz); 3 s non-overlapping epochs were utilized. Mean of all PAC values both within and across channels was used for normalization of PAC value for all epochs across all electrodes, in this case creating a 2400 × 16 matrix. The seizure onset is the first four contacts of right depth electrodes as seen in Figure 1. Note the infrequency of elevations in PAC on those and other channels but note the most significant elevations were on the seizure onset zone channels across this two-hour period. Of the 2400 epochs over which PAC values were calculated for R1, only 32 had PAC values elevated > 2 SD.

PAC was undertaken so as to increase the sensitivity using a modified high-frequency power envelop to lower frequency phase correlation (xcorr in Matlab) (Figure 2). Low and high frequency contents in the signal are extracted using wavelet filters and the instantaneous phase of the low frequency is correlated with the high frequency amplitude to measure PAC. This is tested within a given time epoch and given band. Without any limitations, all bands conceivably can be tested against all others to create a so-called PACgram (Figure 3A). In this cohort, initially no discrimination of bands was performed and simply the possible bands were treated as all between 1 and 250 Hz. Later, specific frequencies for phase and specific frequencies for amplitude were used to constrain the algorithm. For example, in Figure 3 and Figure 4, the low and high frequency ranges utilized were 0.5–30 Hz and 65–175 Hz, which are consistent with the existing literature [17]. Sub-band analysis was then performed based on these results and can be seen in Figure 6. Restricted band analysis (including but not limited to low gamma or LG at 35–55 Hz, high gamma or HG at 65–115 Hz and ripple at 125–175 Hz) yields an aggregate PAC measure for each frequency bin analyzed (2 Hz bins, using wavelet filtering detailed in Supplement), and this aggregate measure is then compared in every epoch within and between electrodes across the dataset. Both 10 and 3 s epoch durations were utilized as seen in Figure 4 (where 10 s epochs were used in delta sub-band analysis in Figure 6).

#### 2.3.3. Seizure Onset Zone

The SOZ electrodes and time of seizures were determined by identifying the elec-trodes with the earliest iEEG seizure discharge, and verified by both a trained epileptolo-gist and a trainee. Seizure onset times and zones were determined by visual identification of a clear electrographic seizure discharge, followed by a look back in the iEEG recordings for the earliest electroencephalographic change concomitant with the seizure [4,50].

#### 2.3.4. Sleep Scoring with Scalp EEG

Behavioral state was determined with scalp EEG signals and scored visually by a neurologist board certified in sleep medicine. All EEG scalp recordings were bandpass filtered 0.3–75 Hz and 60 Hz notch filter for scoring. Visual sleep scoring was in accordance with standard methods (AASM 2012) with modification for replacing the electrooculogram (EOG) recording with FP1, FP2, FPZ scalp electrodes. Wakefulness was determined by the presence of eye blinks visualized in fronto-parietal scalp leads, accompanied by posteriorly dominant alpha rhythms (8–12 Hz) comprising >50% of the epoch. Slow-wave sleep (N3) was scored when high-voltage (>75 μV) delta (0.5–2 Hz) frequency scalp EEG activity was present in at least 20% of the epoch (i.e., at least 6 s within a 30 s epoch) in the frontal derivations using conventional International 10–20 System electrode placements (FP1, FP2, FZ, F3, F4, CZ, C3, C4, O1, O2, and Oz). A similar approach has been used in previous studies [4].

In answering the question of whether there is difference in interictal time irrespective of behavioral state between SOZ and non-SOZ electrodes, simple PACgram analysis was undertaken in every patient at random 1 min periods of time across the two-hour night data segment. As seen in Figure 3A where patient 8 is shown, there was a notable difference. The difference was not consistent across other segments within the same patient nor between patients. Of note however is the range of low frequencies modulating a range of high frequencies in the seizure onset zone electrodes. There appears, qualitatively, to be higher coupling between beta, alpha, and all ranges of gamma (low, high, and low ripple). Figure 3B shows a short segment of this 1 min period and the detected spikes, HFOs, and PAC all overlaid together. Qualitatively, one can see that spikes and HFOs have some correlation with higher PAC values but and that perhaps alpha, beta, and delta are significant low-frequency modulating bands.

## 3. Results

To address the issue concerning temporal variation in the PAC metric (despite somewhat regular IED spiking on SOZ channels in this patient across the whole 2 h), surface plots were generated revealing elevated PAC values across the entire 2 h period. In Figure 4 is shown again patient 7. Notice that R1, R2, R3, and R4 were clinically determined as SOZ channels in this patient and indeed high PAC values predominate on those channels. However, also note the infrequency with which these elevated values occur. Calculations were made across a wide modulating band and a wide modulated band to increase sensitivity, but even so only across approximately 1% of the 2 h period in any given channel is elevated PAC observable. Calculations were log normalized and then Z-scored across epochs and channels for the entire period creating a 2400 × 16 matrix in the case of this patient but otherwise a 2400 × N matrix in all other patents.

**Figure 5:**
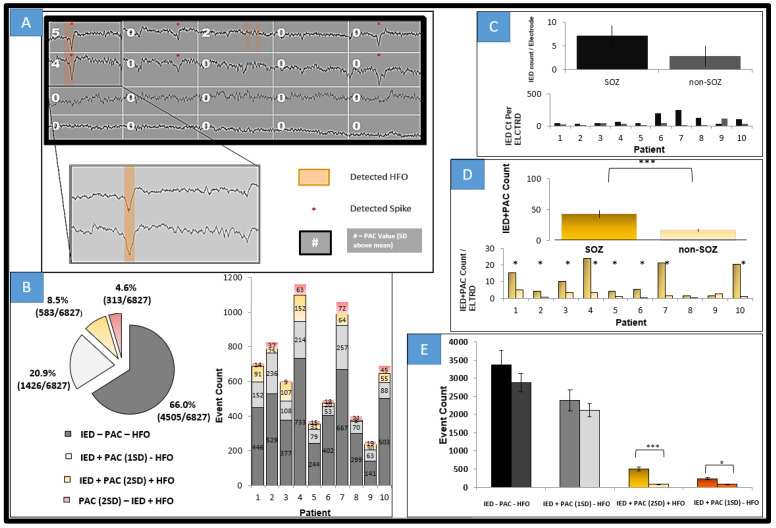
Event counts comparing HFO, PAC, and IEDs. (**A**) 15 s segment from four channels in patient 2; the top 2 channels are SOZ. PAC values in SD (mean of all electrodes across a 2 h period) rounded to nearest integer for 3 s segments frequency for phase 0.5–30 Hz, frequency for amplitude set at 65–175 Hz. Note the highest PAC values are for those where IEDs (denoted by *) and HFOs (orange highlight) are co-incident. In the first channel, third epoch, note two HFOs were detected and PAC was elevated 2 standard deviations above the mean. Notice also in the second and fifth epoch of first channel that spikes are detected with no significant elevation of PAC. (**B**) Total event counts across 2 h epochs all channels 10 patients with breakdown by patient on the right. IED alone constituted the bulk of detected events. IEDs and PAC values at least >2 SD (log-normalized as most values were near 0), were the next most prevalent. The next most prevalent event type was an elevated PAC epoch with a detected HFO (as seen in channel 1 epoch 3). IED within 50 ms of a detected HFO was the next most prevalent event type. Elevated PAC epoch nearly always included either a detected spike or HFO. (**C**) Avg Spike count per patient (non-PAC and non-HFO IEDs) in SOZ and non-SOZ all patients as well as per patient breakdown. Consistent with other results, IEDs are increased within SOZ among all but 1 patients (pt9 which has a low spike count). SEM shown. (**D**) IED + PAC counts on all and individual patient electrodes of PAC positive spikes, *** = *p* < 0.001 comparing SOZ to non-SOZ. This shows that PAC, particularly with spikes discriminates SOZ interictally. (**E**) Event count as seen in B broken down by event type and into SOZ and non-SOZ (as indicated by the double bar for each type of event). * *p* < 0.05 *** *p* ≤ 0.001.

Interictal epileptiform spiking is seen with much higher regularity, although correlated and seen in the SOZ. A great deal of the associated elevations in wide-spectrum (0.5–30 modulating 65–175 Hz) PAC (here defined as >2 SD across the entire 2 h period and assessed across all channels) occurred with IEDs. Since 3 s segments were used to calculate PAC values, sometimes 2 IEDs were within a single epoch although in a small minority of observations. These 3 segments were not included in Figure 5. Figure 5A illustrates in patient 2, who had bilateral MTS, four example channels and 15 s of analyzed data. Normalized PAC values rounded to the nearest integer are displayed along with detected spikes and detected HFOs. PAC elevations are seen with IEDs and with HFO, although to a greater extent with spikes. However, some IEDs are not associated with elevations in PAC. In fact, from Figure 5B which displays all detected events in every channel across all 10 patients, most detected IEDs were not associated with elevated PAC values or with HFOs for that matter. A minority of IEDs are associated with elevated PAC values and HFOs. Figure 5C examines at the group and individual levels the average spike count per patient, grouping all SOZ and non-SOZ electrodes together. At this level there is not a statistically significant effect in mean differences, but if the average count is taken per electrode within a single patient, a significant difference in means was noted (*p* < 0.005). Subjects on average had 15 (SEM 0.15) electrodes and SOZ electrode counts of 3 (SEM 0.71). When analyzing IED + elevated PAC, grouped SOZ electrodes among patients and within individuals as shown in Figure 5D show a strongly significant difference in means (*p* < 0.001), also when taking into account electrode numbers within each group (*p* < 0.0001). At the individual level, significance of mean differences (*p* < 0.05) is seen for all but one patient (patient 9, see bottom of Figure 5D) in this cohort. Figure 5E is a summary grouping all patients and compares at the total event count level for five types of events (for IED, IED without PAC and without HFO, IED with PAC elevation, IED without PAC elevation but with HFO, and PAC with HFO but without IED), and even at this crude grouping, it is clear that IED + PAC has statistically significant difference in means of SOZ from non-SOZ groups (*p* < 0.001).

**Figure 6:**
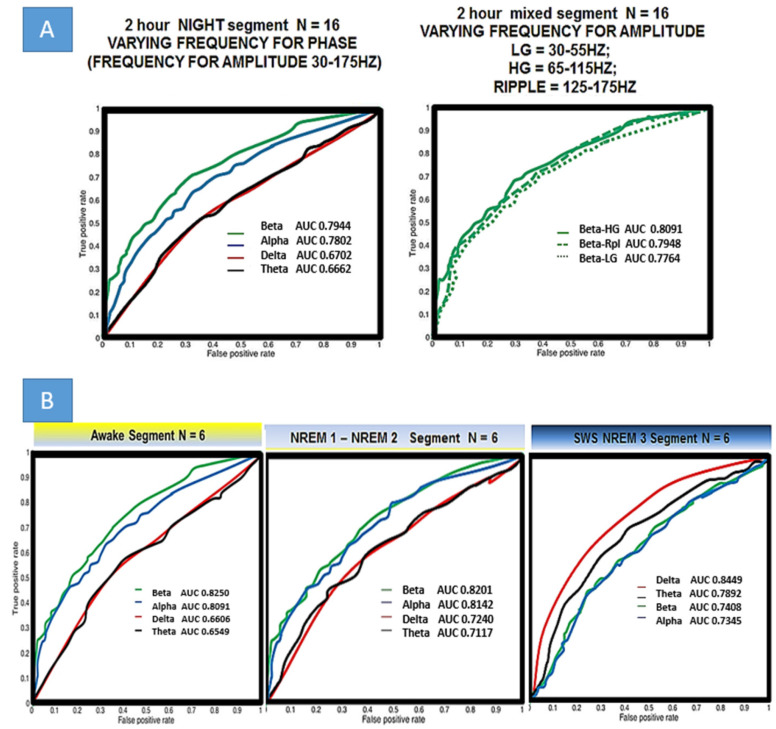
Localization of epileptogenic seizure onset zone by different frequency bands for phase and amplitude. (**A**) SOZ was determined from phase II monitoring and determined by a trained epileptologist. *Left* Holding constant the frequency for amplitude to include all high activity (30–175 Hz), beta is the best localizing (not statistically significant from alpha) band. *Right* Holding the frequency for phase, constant, there is no statistical difference between low gamma LG, high gamma HG, and ripple in terms of SOZ localizing and thus analysis in part B uses wide band HFA. (**B**) Examining different frequencies for phase in varied behavioral/sleep states. Please note that REM was not analyzed. Sleep staging was done by a trained neurologist. 12 min awake segments were used in all ten patients with both internal leads and scalp. EEG Localization is best when delta is the frequency band used to calculate PAC in slow wave sleep/NREM3.

Seeming that elevations in PAC strongly correlate with epileptogenic electrodes, receiver operator curves (ROC, which itself is a reflection of sensitivity and specificity) were generated using linear step equivalent to varying threshold values for PAC in a range of <0,10> STD (normalized as in Figure 4) to generate the ROC plots (Figure 6A,B). True positives, defined as the electrodes which were grouped above the PAC threshold that also were deemed SOZ by epileptologists, were plotted vs. false positives, defined as the number of non SOZ electrodes grouped above PAC threshold. The quality of classification was assessed using area under the receiver operator curve (AUROC). The first ROC in Figure 6A assesses relevance for localization of all of the Berger bands within the wide modulating band (but holding the modulated band constant from 30–175 Hz) used for PAC analysis in Figure 2, Figure 4 and Figure 5. There is a clear difference in this scheme when looking across the entire 2 h segment. Beta modulating low-gamma (30–60 Hz), high-gamma (60–100 Hz), and ripple (100–175 Hz) PAC values resulted in the highest AUC and thus best discriminated SOZ from non-SOZ which in these ten patients was 0.7944. Comparing to AUCs of all patients with the paired *t*-test, there is a statistical difference for means in beta-GammaRipple and alpha-GammaRipple vs. delta-GammaRipple, and beta-GammaRipple and alpha-GammaRipple vs. theta-GammaRipple in terms of discriminating SOZ from nonSOZ (*p* < 0.05). However, there was not a statistical difference of AUC means between beta-GammaRipple versus alpha-GammaRipple and theta-GammaRipple compared to delta-GammaRipple in these ten patients across an entire 2 h night segment of mixed behavioral states (*p* > 0.05). Focusing on the right plot of Figure 6A, holding beta and varying Low-Gamma, High-Gamma, and Ripple, there is no statistical difference in AUCs’ means between the groups. This analysis was carried out for all other modulating bands (delta, theta, and alpha) and held true for all. In Figure 6B, six patients who had fully scored sleep segments were analyzed in the same manner but taking equal size segments of awake, N1-N2, and SWS/N3. The same pattern holds in awake and N1/N2 as is seen in Figure 6A but not in N3. In N3 delta modulation of wide bands of high frequency activity becomes most relevant in discriminating SOZ from non SOZ with AUC 0.8489 significantly different (*p* < 0.05) when compared to other bands.

## 4. Discussion

Variation of epileptic activity is known to occur in different sleep states. During REM sleep for example, it has been shown that IEDs and HFOs are less likely to occur compared to non-REM sleep [51]. It is also known that slow wave sleep is a period of time where brain communication occurs using slow oscillations and higher frequency activity [52]. There is more and more focus on this as a biomarker for pathological tissue in the study of epilepsy and other brain diseases [27].

Our results show that PAC in this small cohort correlates highly with SOZ in medication of refractory epilepsy and may be a useful biomarker of epileptogenic tissue. This is consistent with the previous studies [12,13,18,27,28,40,53,54,55], but is unique in several aspects. While other studies have shown an increase in PAC during SWS, this fact was not used to evaluate sensitivity and specificity of localization in any size cohort. This may be because sleep-scoring is difficult in intracranial patients, but hopefully with the advent of automated intracranial methods for sleep scoring this will not be such a limitation in the future [4].

Next, there has been a great deal of focus on PAC within the ictal or immediate pre-ictal period [12,18,54], but less focus on the interictal time. In the literature there is discussion of neural activities in epilepsy and there is some consensus into interictal, ictal, and postictal classification. While pre-ictal states consistently herald seizure onset within seconds or minutes, their pro-ictal counterparts signify variations in neural activity across lengthier time scales, during which a propensity for seizures exists. The presence of (pathologic) pro-ictal states among a plethora of otherwise physiologic (e.g., sleep–wake cycle) is the subject of recent inquiry although many variables likely exist beyond behavioral state in this analysis. Of course, for this work to have the most relevance, ultimately not only interictal electrophysiologic biomarker analysis must be performed but anesthetized behavioral states would also need to be considered. iEEG has dramatically different patterns in such states and presumably so would PAC. Investigations in animals using fMRI have yielded some interesting results [43,44], but further work and good data sets are needed [56,57]. Some studies suggest that PAC may be related to brain states and change in anesthesia states [50,56,57,58].

It is not clear whether the PAC values found in these patients are comparable to other brain regions and thus should not be taken as a general biomarker of SOZ without also taking into account brain region as it has been shown that HFOs vary with brain region [59]. Additionally, the nature of these recordings is that placement of the arrays is along the axis of the hippocampus and other mesial temporal structures, which makes it less clear whether even the results could translate to lateral cortex of the same lobe. Further experiments are needed to take into account these anatomical variations and should aim to explore a more heterogeneous patient population. What is encouraging is that this cohort represents a wide age range (27–70 yo) with age of onset also widely ranged (3–52 yo) and both left and right focal epilepsy are represented in this cohort.

A study by Amiri has an interesting overlap with this study. They note that coupling intensity (note that absolute intensity was not measured in this study), was highest during N3 sleep and lowest during REM (note assessed here as too few segments across patients). Most notably, PAC was at a higher absolute level in SOZ compared with non-SOZ during N3. Their results suggest that elevated PAC might reflect some basic electrophysiologic disturbance in the interictal and ictal times. It has been suggested by others that ripples on spikes are key markers of focal epilepsy [18]. It should be noted that Amiri did not however look further into the localizing value, as was done in this study, even though different Berger Bands were examined. A study by Edakawa found that seizure onset time can be identified using PAC but not with the spatial location [13]. even though here we see it can. Edakawa suggested though that even though they were interested in pre-ictal and ictal times and found that beta-GammaRipple PAC was most discriminating of the temporal onset of preictal and ictal states, that similar patterns might emerge during certain periods of interictal time. This study corroborates that finding outside of slow wave sleep but importantly the best discriminating value for seizure onset zone in this study occurs during slow wave sleep (N3) using delta as the frequency for phase irrespective of the frequency for amplitude (so long as in the high frequency range). One explanation for this may simply be that more physiologic coupling occurs in these ranges during these respective behavioral states.

Although IED + PAC was found more frequently in SOZ, the relationship between both and seizures should be further explored. At this point there is some interesting work being undertaken with HFOs on spikes which may help explain this phenomenon [18]. The relationship between HFOs not associated with spikes, while it does not appear to provide SOZ localizing value, is also being studied. Further research will help explain this and should help find the precise relationships between these processes and the necessary treatments which might help alleviate them.

It should be said that 2 h time segment analysis is somewhat arbitrary but another analysis focused on this showing that similar results can be obtained with even shorter time segments in the range of 20 min [8,9,42].

Ultimately, the clinical significance of interictal biomarkers can be determined by their association with seizure-free outcomes. In this study, we did not find a significant difference comparing PAC localization and surgery outcomes. This would also be a good direction for future research but would be appropriate for a much larger cohort than is presented here.

## Supplementary Materials

The following supporting information can be downloaded at: https://www.mdpi.com/article/10.3390/life13051186/s1, Supplement File S1: Sleep Scoring of iEEG with scalp EEG and Clinical Profile of Patients; Supplement File S2: Interictal Spike and HFO Detection of iEEG. Table S1: Clinical Profile of 17 patients with medically refractory epilepsy. Reference [60] is cited in Supplementary Materials File.

## Appendix A. Phase-Amplitude Coupling

In order to investigate the cross-frequency coupling interactions, we simply looked at the correlation between a low frequency signal and the time-course of the power at higher frequencies. Let the signal {Xt} be represented by the time series X1, X2, …, XN. First, the time-course of power was estimated for frequency f2 by applying a sliding tapered time-window followed by a Shannon Wavelet represented by the analytic expression here expressed in series notation:

P_{t}(f_{modulated}) = 1/\Delta t\left | \sum_{t = 0}^{n + 1}\ \gamma X_{t-\frac{n}{2}}e^{-i2\pi f_{ modulated}1/\Delta t} \right |^{2}  (A1)

A Hanning taper n points is the length of the sliding time window. Next, the correlation xcorr(fmodulating,fmodulated) was estimated between signal {Xt} and the estimate of the time-course of power{Pt(fmodulated)} at a given frequency fmodulating:

The correlation was the absolute value of the correlation. The phase difference between the signal at fmodualting and the power at fmodulated is given by the angle. In this case refer to a 2048 points Hanning window and * to the complex conjugate. This allowed us to characterize the phase-to-power cross-frequency interaction with respect to f and fmodulated spectral bin by spectral bin and time epoch by time epoch [27].
