# Supplementary material for: Phase-Amplitude Coupling Localizes Pathologic Brain with Aid of Behavioral Staging in Sleep
Source: Life (Basel). 2023 May 15;13(5):1186. doi: 10.3390/life13051186 (PMC10221792; doi:10.3390/life13051186)
Supplement: Supplementary file 1 [file life-13-01186-s001.zip › life-2280277-supplementary-highlight.pdf]

Supplement File S1. Sleep Scoring of iEEG with scalp EEG and Clinical Profile of Patients

Behavioral state was determined with scalp EEG signals and scored visually by a neurologist board certified in sleep medicine (EKS). All EEG scalp recordings were bandpass filtered 0.3–75 Hz and 60 Hz notch filtered for scoring. Visual sleep scoring was in accordance with the standard methods [1] with modification for replacing the electrooculogram (EOG) recording with FP1, FP2, FPZ scalp electrodes. Wakefulness was determined by the presence of eye blinks visualized in fronto-parietal scalp leads, accompanied by posteriorly dominant alpha rhythms (8–12 Hz) comprising >50% of the epoch. Slow-wave sleep (N3) was scored when high-voltage (>75 uV) delta (0.5–3 Hz) frequency scalp EEG activity was present in at least 20% of the epoch (i.e., at least 6 s within a 30 s epoch) in the frontal derivations using conventional International 10-20 System electrode placements (FP1, FP2, FZ, F3, F4, CZ, C3, C4, O1, O2, and Oz). A similar approach has been used in previous studies [2,3].

Table S1. Clinical Profile of 17 patients with medically refractory epilepsy. Per IRB approved protocols, and recorded during their Phase II Monitoring in preparation for resection surgery, 17 patients were studied. Key demographic information is noted in the table. Right. F—focal seizures, G—Generalized tonic-clonic seizures. RAT—right anterior temporal. LAT—left anterior temporal. LLT—left lateral temporal. RAI—right anterior insula. RF—right frontal. ILAE 1—seizure and aura free, ILAE 6—significantly worse than before surgery. SOZ electrodes varied between 2 and 12. G,S,D—grid, strip depth electrodes.

Subject	Age of Onset	Handedness	Sex	Age	Phenotype	SOZ	Channel Count	Electrode Types	MRI	Surgery Outcomes	Notes
1	52	R	F	58	F	RAT, LAT	16	B/L Occ. Depth	B/L Hip Atrophy	NA	VNS
2	29	R	F	70	F(C)	RAT, LAT	15	B/L Occ. Depth	Normal	NA	Bilateral SOZ
3	14	R	F	35	F(C)	RAT, LAT	15	B/L Occ. Depth	Normal	NA	No resection, b/l SOZ
4	37	R	M	46	F(C), G	LLT	16	B/L Occ. Depth	L. Temp. angioma	NA	Eloquent SOZ
5	3	R	F	27	F(C), G	RAT, LAT	16	B/L Occ. Depth	B/L MTS	NA	VNS
6	18	L	M	38	F(C)	LAT	15	B/L Occ. Depth	B/L periventricular heterotopia	ILAE 1	LAT lobectomy
7	41	R	M	61	F(C), G	RAT, RF	16	B/L Occ. Depth	Normal	ILAE 2	RAT lobectomy
8	4	R	F	32	F(C), G	RAT	15	B/L Occ. Depth	B/L MTS	ILAE 6	RAT lobectomy
9	39	R	M	47	F(C)	RT, LT	16	B/L Occ. Depth	Pineal cyst, B/L Hip atrophy	NA	B/L SOZ
10	3	R	F	22	F(C), G	LAT	16	B/L Occ. Depth	B/L MTS	ILAE 1	LAT Lobectomy
11	12	R,L	F	19	F(C), G	LAT	56	G,S,D	L. Hip atrophy	ILAE 1	LAT Lobectomy

12	16	R	M	26	F(C)	RAI	123	G,S,D	R insula encephalomalacia	NA	Subthresh Stim
13	22	R	M	34	F(C)	RF	88	G,S,D	Posterior temp. lesion	ILAE 1	RF corticect
14	14	R	M	23	F	RF	154	sEEG	Nonlesional	NA	VNS
15	6	R	M	56	F	RF, RT	88	G,S,D	RF and RT encephalomalacia	NA	Subthresh Stim
16	18	R	F	34	F	LAT	32	G,S,D	L. MTS	NA	L. Temp. Lobectomy
17	14	R	M	33F	F	LT	76	G,S,D	LAT atrophy	ILAE 2	Neocortical Resection

Supplement File S2: Interictal Spike and HFO Detection of iEEG

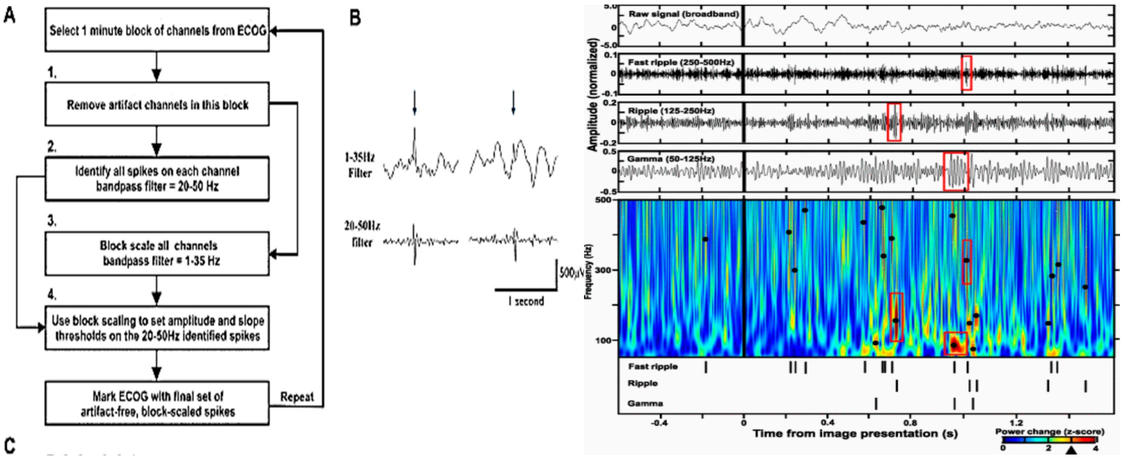

(A) The spike detection algorithm [4], extracts successive 1 min blocks of iEEG and removes artifact channels. Individual channels were defined as average slope greater than 10 SD outside of mean slopes of all channels. Second, iEEG was bandpass filtered 20–50 Hz to identify possible spikes, where a sharp discharge must last between 20 and 70 ms. Absolute amplitudes of peaks greater than 4SD of channel mean amplitude were noted as potential spike locations for further consideration. Third, raw iEEG was bandpass filtered (second order Butterworth) 1–35 Hz. A scaling factor is determined by finding a value that will bring the median of all channel amplitudes to 70 µV. All channels are multiplied by this scaling factor. Once the data have been scaled, the amplitude and slope of each half-wave of the potential spikes identified previously in step 2 are calculated and the values are compared to static thresholds (total amplitude of both half-waves > 600 µV, slope of each half-wave > 7µV/ms, duration of each half-wave > 10 ms). Potential spikes with half-waves that exceed these thresholds are marked as interictal spikes. (B) HFOs were detected using a Hilbert transform-based method, as previously reported [3]. Transformation of continuous HFO signals to point processes. Example of a gamma, ripple, and fast ripple HFO detections recorded on one macro-contact in temporal lobe. Red boxes highlight the three detections in the filtered recording traces (top), and their corresponding power changes in the spectrogram (middle). Black dots indicate all HFOs detected in this signal, which are also marked in the raster plot (bottom) broken down into gamma, ripple, and fast ripple point processes. Notice the discrete duration and frequency signature of each HFO detection that was used to transform it into point-process detections at the time of peak power.

1. Iber C, Ancoli-Israel S, Chesson A, Quan SF. for the American Academy of Sleep Medicine. The AASM Manual for the Scoring of Sleep and Associated Events: Rules, Terminology and Technical Specifications. American Academy of Sleep Medicine; Westchester, IL: 2007.
2. Kremen, V.; Duque, J.J.; Brinkmann, B.H.; Berry, B.M.; Kucewicz, M.T.; Khadjevand, F.; Van Gompel, J.; Stead, M.; Louis, E.K.S.; Worrell, G.A. Behavioral State Classification in Epileptic Brain Using Intracranial Electrophysiology. *J. Neural Eng.* **2017**, *14*, 26001. https://doi.org/10.1088/1741-2552/aa5688.
3. Kucewicz, M.T.; Berry, B.M.; Bower, M.R.; Cimbalnik, J.; Svehlik, V.; Stead, S.M.; Worrell, G.A. Combined Single Neuron Unit Activity and Local Field Potential Oscillations in a Human Visual Recognition Memory Task. *IEEE Trans. Biomed. Eng.* **2016**, *63*, 67–75. https://doi.org/10.1109/TBME.2015.2451596.
4. Barkmeier, D.T.; Shah, A.K.; Flanagan, D.; Atkinson, M.D.; Agarwal, R.; Fuerst, D.R.; Jafari-Khouzani, K.; Loeb, J.A. High Inter-Reviewer Variability of Spike Detection on Intracranial EEG Addressed by an Automated Multi-Channel Algorithm. *Clin. Neurophysiol.* **2012**, *123*, 1088–1095. https://doi.org/10.1016/j.clinph.2011.09.023.
